# Reduced Scaling Correlated Natural Transition Orbitals for Multilevel Coupled Cluster Calculations

**DOI:** 10.1021/acs.jpca.4c06271

**Published:** 2024-10-24

**Authors:** Sarai Dery Folkestad, Henrik Koch

**Affiliations:** Department of Chemistry, Norwegian University of Science and Technology, NTNU, 7491 Trondheim, Norway

## Abstract

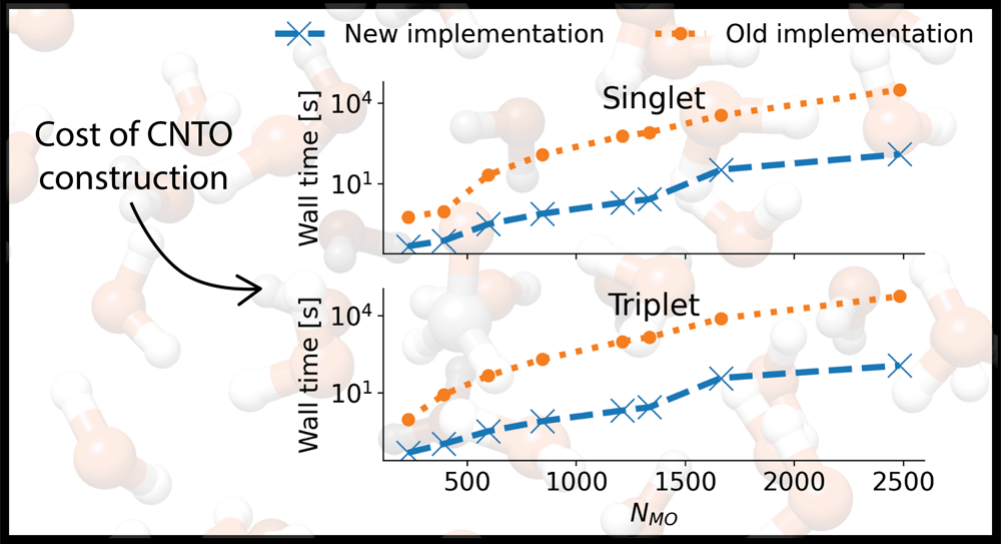

Multilevel coupled cluster theory offers reduced scaling computation of intensive properties in systems that are too large for standard coupled cluster calculations. A significant benefit of the multilevel coupled cluster framework is the possibility of calculating intensive properties that are not tightly localized if an appropriate set of active orbitals is used. Correlated natural transition orbitals (CNTOs) are tailored to describe excitation processes. For multilevel coupled cluster singles and doubles (MLCCSD) and singles and perturbative doubles (MLCC2) calculations, the construction of CNTOs generally becomes the computational bottleneck. Here, we demonstrate how CNTOs can be obtained with  operations, eliminating the -scaling steps involved in the original approach. This reduction in scaling moves the bottleneck of MLCC2 and MLCCSD calculations from the active orbital space preparation to the MLCC2 and MLCCSD equations with -scaling.

## Introduction

The steep polynomial scaling of the standard coupled cluster models^1^ has long motivated the development of reduced scaling approximate coupled cluster approaches. The approaches can be divided into methods that aim to accurately describe total energies^2−13^ and methods that aim to model intensive properties of a target region.^14−18^ Multilevel coupled cluster theory^19,20^ is among the last group of methods.

In multilevel coupled cluster theory,^20^ high-order excitations in the cluster operator are restricted to an active orbital space, effectively reducing the scaling to that of a lower-level coupled cluster model. For instance, in multilevel coupled cluster singles and perturbative doubles (MLCC2), or multilevel singles and doubles (MLCCSD), the target accuracy is that of the CC2 and CCSD models. At the same time, the cost approaches that of a coupled cluster singles (CCS) calculation.

The accuracy and computational savings of multilevel coupled cluster calculations rely on the choice of active orbitals, i.e., the orbital type and active space size. If the intensive property of interest is confined to a small region of the molecular system, localized orbitals, such as Foster-Boys,^21^ Pipek-Mezey,^22^ Edmiston-Ruedenberg,^23^ Cholesky,^24,25^ or projected atomic orbitals,^26,27^ are viable choices. To model excitation processes that are not tightly confined to a region of the molecule, however, localized orbitals will not give a sufficiently compact active orbital space and their use also relies on *a priori* knowledge of the character of the excitation process. In such cases, a compact active space can be constructed by exploiting the information available about the excitation process obtained at a lower level of theory. For this purpose, we use the correlated natural transition orbitals (CNTOs) introduced by Ho̷yvik et al.^28^

CNTOs are constructed by a singular value decomposition of the excitation vectors from a correlated calculation. The matrices of singular vectors transform the orbitals from the original molecular orbital basis to the CNTO basis. The excitation vectors used to generate the CNTOs must include, at least approximately, double excitations in the parametrization. The obvious candidate models to obtain these excitation vectors are the CC2, CCSD, or as Baudin and Kristensen^29^ have suggested, the CIS(D) models. These vectors are therefore obtained with, at least, an  cost (iterative for CC2 and one-shot for CIS(D)).

Previously, we have constructed CNTOs using the prescription of Baudin and Kristensen^29^ to calculate MLCC2 and MLCCSD singlet^30^ and triplet^31^ excitation energies. However, the asymptotic scaling of the MLCC2 and MLCCSD equations is , and the one-shot  step to prepare the CNTOs will become the bottleneck. In this paper, we construct approximate excitation vectors in a reduced occupied space. Thereby reducing the overall cost of the CNTOs to .

### Theory

The coupled cluster wave function is given by
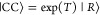
1where *T* is the cluster operator,
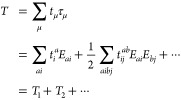
2that generates excitations of the reference determinant |R⟩. We use the spin-adapted closed-shell formulation of coupled cluster theory^1^ where the *E*_pq_ operators generate singlet excitations from orbital *q* to orbital *p*. The cluster operator can be organized according to excitation order, e.g., the operators in *T*_1_ excite a single electron, the operators in *T*_2_ excite two electrons, and so on. We adopt a notation where *i*, *j*, *k*, ··· denote orbitals occupied in the reference determinant, and *a*, *b*, *c*, ··· denote virtual orbitals. General orbitals are denoted by indices *p*, *q*, *r*, *s*, ··· The coupled cluster ground state is obtained by solving the projected Schrödinger equation and the excited states are calculated using coupled cluster linear response theory^32^ or equation-of-motion coupled cluster theory.^1,33^ The standard models of coupled cluster theory are defined by the truncation level of the cluster operator. For instance, in coupled cluster singles and doubles (CCSD), where the cluster operator is defined as *T* = *T*_1_ + *T*_2_. Furthermore, perturbative approaches exist that include higher-order excitations using perturbation theory. An important example is the coupled cluster singles and perturbative doubles^34^ (CC2) method.

### Multilevel CC2 and CCSD

In multilevel coupled cluster theory, the *t*-amplitudes of the higher-order excitations are restricted to an active orbital space. For instance, in MLCC2 and MLCCSD (CCSD–in–CCS),^30^ the cluster operator becomes
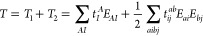
3where upper-case indices refer to the full orbital space, whereas lower case indices are restricted to the active space. The asymptotic scaling of MLCC2 and MLCCSD (for a fixed active space) is that of the lower level CCS model, i.e., the equations scale as , where *N* is a measure of the total system size.^31,35^

### CNTOs for MLCC2 and MLCCSD

The CNTOs are an extension of natural transition orbitals (NTOs), where the matrices
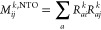
4
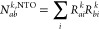
5are diagonalized to obtain orbital transformation matrices. This amounts to a singular value decomposition of the *k*’th excitation vector, *R*^*k*^. To construct NTOs, we use excitation vectors that only include single excitations, e.g., from a CCS (CIS) or TDHF calculation. For CNTOs, we use excitation vectors that also include double excitation contributions. The expressions for the singlet CNTO-matrices are^28^
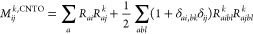
6
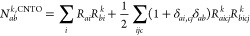
7and for triplet excitations, we have
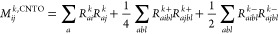
8
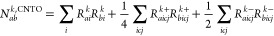
9Here, *R*^*k*+^ and *R*^*k*–^ are the doubles amplitudes of the triplet excited state, see ref (36). Excitation vectors that include double excitations can be obtained from CC2 or CCSD calculations. Hence, the use of CNTOs is seemingly restricted to use in MLCC3 or higher orders of multilevel coupled cluster theory. However, Baudin and Kristensen demonstrated how approximate CNTOs could be obtained using CCS (CIS) excitation vectors only.^29^ They proposed the construction of approximate double excitation vectors, according to the CC2 expressions (i.e., CIS(D) excitation vectors).^37,38^ These approximate CNTOs have been used to calculate singlet excitations within the multilevel coupled cluster framework,^30^ and recently, we used the same procedure to obtain active spaces for triplet excited states.^31^ For singlet and triplet excited states, we have
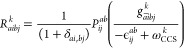
10and
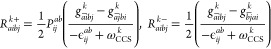
11respectively. Here, ω^CCS^ is the CCS excitation energy, and ϵ_*ij*_^*ab*^ = ϵ_*a*_ + ϵ_*b*_ – ϵ_*i*_ – ϵ_*j*_, where the ϵ_*p*_ are orbital energies. We have also defined transformed two-electron integrals,
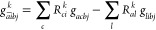
12in terms of the singlet or triplet CCS excitation vector, *R*^*k*^, and the electron-repulsion integrals, *g*_*pqrs*_.

The ***M***^CNTO^ and ***N***^CNTO^ matrices (suppressing the superscript *k*) are diagonalized and the eigenvectors are used to transform the occupied and virtual orbitals, respectively, to the CNTO basis. The eigenvalues can be used to choose the number of orbitals in the occupied space.^28^ We introduce two thresholds (δ_o_ and δ_v_) and select the minimal number of orbitals for which
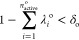
13
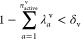
14where the eigenvalues of ***M***^CNTO^ and ***N***^CNTO^ (λ_*i*_^o^ and λ_*a*_^v^) are added in decreasing order.

The CNTOs offer a compact orbital space for multilevel coupled cluster calculations. However, both constructing the approximate double excitation vectors and the ***M***^CNTO^ and ***N***^CNTO^ matrices, entail -scaling operations, making the CNTO-construction the bottleneck of MLCC2 and MLCCSD calculations. The NTOs, on the other hand, can be constructed in -scaling operations. Unfortunately, from eq 5, we see that the rank of ***N***^NTO^ cannot exceed the number of occupied orbitals, *n*_o_. The ranks of the ***M*** and ***N*** matrices restrict the number of meaningful occupied and virtual transition orbitals. The importance of a CNTO is given by the magnitude of the corresponding eigenvalue. Generally, we need an active virtual space that exceeds *n*_*o*_ in size. Therefore, NTOs do not contain sufficient information for virtual orbital space selection.

To avoid the -scaling step of the CNTO construction, we exploit that the occupied NTOs, in contrast to the virtual NTOs, provide an appropriate set of active occupied orbitals. We can therefore use ***M***^NTO^ to generate a reduced occupied orbital space within which we generate the approximate double excitation vectors needed for the construction of ***N***^CNTO^. The restriction of the occupied indices in eqs 6–12, reduces the scaling of the approximate CNTOs to  in the asymptotic limit.

## Results and Discussion

The approximate CNTOs have been implemented in a development version of the e*^T^* program.^41^ We use the aug-cc-pVDZ basis in all calculations and the frozen-core approximation is applied throughout. Default convergence thresholds of e*^T^* v1.9.x are used to solve all equations: the Hartree–Fock equations are solved with a residual threshold (maximum norm) of 10^–7^ a.u., Cholesky decomposition^42^ of the electron repulsion integrals is performed with a threshold of 10^–4^ a.u., and the coupled cluster ground and excited state equations are solved to residual thresholds (*l*_2_-norm) of 10^–5^ a.u. and 10^–3^ a.u., respectively. Timings have been performed on an Intel Platinum 8480 node, using 56 threads and 1 TB of memory available.

**Figure 1:**
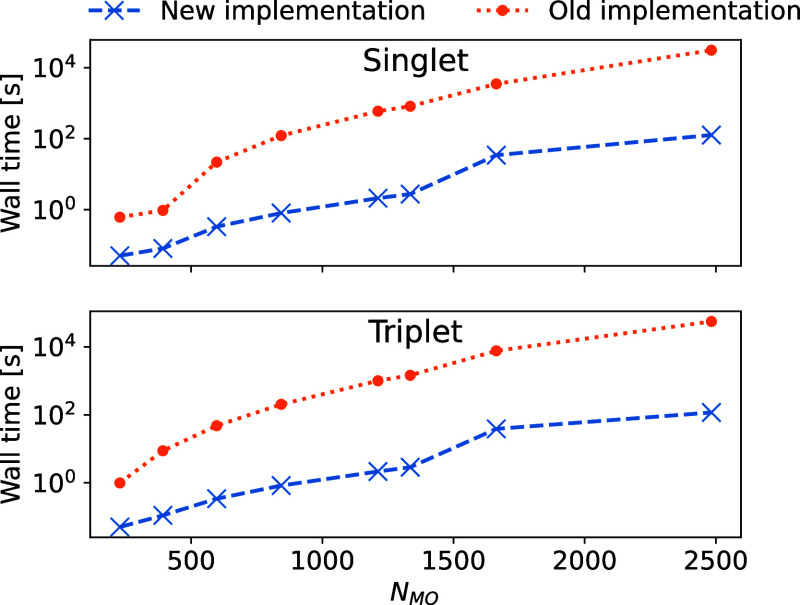
Wall time to construct approximate CNTOs (excluding the time to generate the CCS excitation vectors) from full space approximate doubles (old implementation) and from reduced space approximate doubles (new implementation). The upper panel shows the singlet case, the lower panel shows the triplet case. Note that the plot is on a logarithmic scale.

In Figure 1, we show wall time comparisons for the singlet and triplet CNTO construction of a formaldehyde molecule surrounded by (4–59) water molecules. The geometries can be found in ref (43). Note that the plots are on a logarithmic scale. Increasing the number of water molecules, the number of molecular orbitals of the system increases, and, with it, the cost to generate the CNTOs. We observe significant cost reduction with the reduced space CNTOs, in excess of 2 orders of magnitude for formaldehyde and 59 water molecules.

**Figure 2:**
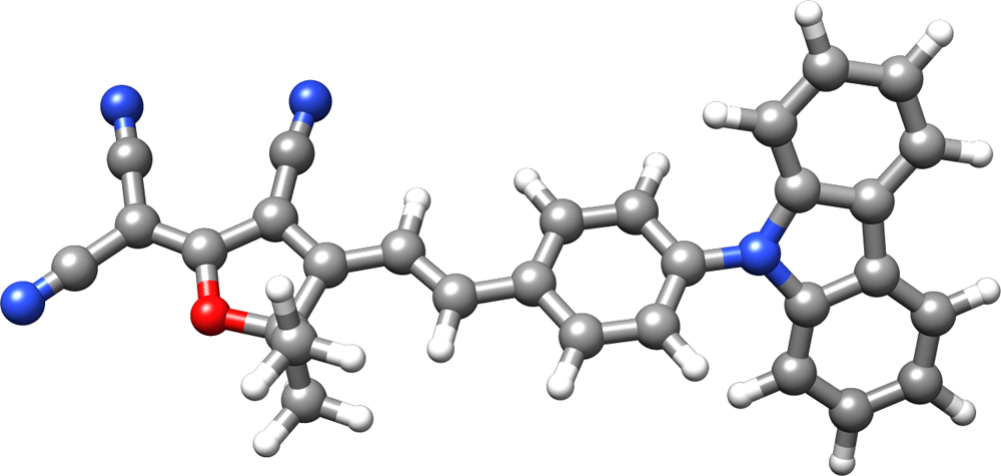
CzTDF molecule^39^ exhibits thermally activated delayed fluorescence, Here, it is used for the calculations presented in Figures 3 and 4, and Tables 1 and 2. Geometry can be found in the Supporting Information of ref (40).

**Figure 3:**
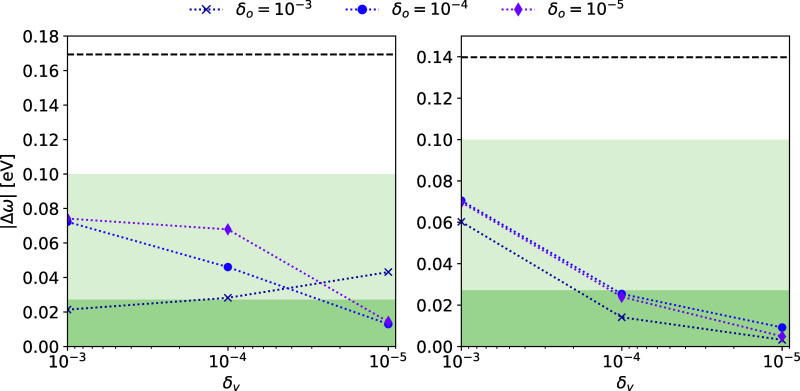
Absolute errors of the lowest triplet MLCC2 (left) and MLCCSD (right) excitation energies |Δω| with CNTO thresholds. Each line corresponds to an occupied threshold δ_*o*_ and the virtual threshold is on the *x*-axis. Shaded areas indicate the 0.1 eV and 1 m*E*_h_ deviations from the CC2 and CCSD energies. The dashed line indicates the error of the lower-level method in the multilevel calculation, i.e., CCS.

**Figure 4:**
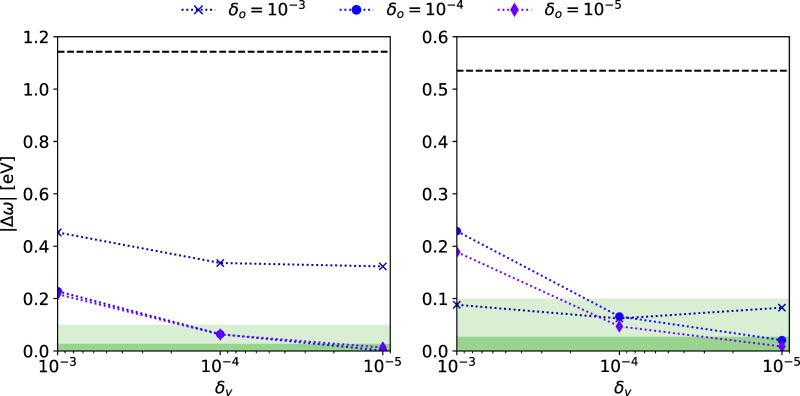
Absolute errors of the lowest singlet MLCC2 (left) and MLCCSD (right) excitation energies |Δω| with CNTO thresholds. Each line corresponds to an occupied threshold δ_o_ and the virtual threshold is on the *x*-axis. Shaded areas indicate the 0.1 eV and 1 m*E*_h_ deviations from the CC2 and CCSD energies. The dashed line indicates the error of the lower-level method in the multilevel calculation, i.e., CCS.

For the molecule in Figure 2 (CzTDF^39^), we consider the convergence of the lowest MLCC2 and MLCCSD excitation energies toward the CC2 and CCSD excitation energies. The geometry can be found in the Supporting Information of ref (40). The results are shown in Figures 3 and 4. As both thresholds δ_*o*_ and δ_*v*_ are tightened, the multilevel coupled cluster excitation energies tend toward the excitation energies of the target coupled cluster method. The MLCC2 energies are more sensitive to the CNTO thresholds, as has previously been observed for CNTOs using full-space approximate double excitation vectors. Nevertheless, errors within 0.1 eV are obtained for δ_o_, δ_v_ ≤ 10^–4^.

In Tables 1 and 2, we compare the old (full-space) and new (reduced-space) implementations of the approximate CNTOs for the five lowest excited states of CzTDF. CNTO thresholds of δ_o_ = δ_v_ = 10^–4^ are used. The excitations have different character, some are delocalized over the entire molecule, and some are more localized. The dominant CCS NTOs for these excitations can be found in the Supporting Information. Compared to the corresponding standard coupled cluster models (CC2 and CCSD), the mean average error (MAE) is always below 0.05 eV. However, because of the larger active spaces, the errors of the old CNTO approach are lower. For the same reasons, the cost of the MLCC calculations is higher using the old approach, even when we disregard the cost of generating the CNTOs. The cost to construct the CNTOs has been reduced by a factor of ∼5 in the singlet case and ∼3 in the triplet case.

**Table 1:** Five Lowest MLCC2/aug-cc-pVDZ and MLCCSD/aug-cc-pVDZ Triplet Excitation Energies of CzTDF, Compared to the Standard Coupled Cluster Models^a^

model	version	ω_1_	ω_2_	ω_3_	ω_4_	ω_5_	MAE
MLCC2	old	2.153	2.816	3.369	3.627	3.722	0.015
	new	2.173	2.924	3.490	3.678	3.808	0.088
CC2		2.152	2.802	3.354	3.594	3.733	
MLCCSD	old	2.120	3.008	3.468	3.623	3.816	0.005
	new	2.119	3.045	3.466	3.677	3.840	0.021
CCSD		2.122	3.012	3.474	3.626	3.828	

a Mean absolute error is also reported. With full space approximate doubles, the resulting active space contains *n*_o_ = 77 occupied and *n*_v_ = 386 virtual orbitals. With reduced space approximate doubles, the resulting active space contains *n*_o_ = 71 occupied and *n*_v_ = 331 virtual orbitals. In total, the molecule has 83 occupied orbitals and 866 virtual orbitals (excluding the frozen core orbitals).

**Table 2:** Five Lowest MLCC2/aug-cc-pVDZ and MLCCSD/aug-cc-pVDZ Singlet Excitation Energies of CzTDF in eV, Compared to the Standard Coupled Cluster Models^a^

model	version	ω_1_	ω_2_	ω_3_	ω_4_	ω_5_	MAE
MLCC2	old	2.687	3.506	3.641	4.045	4.124	0.014
	new	2.719	3.537	3.643	4.076	4.146	0.036
CC2		2.687	3.481	3.638	4.048	4.087	
MLCCSD	old	3.292	4.029	4.269	4.444	4.455	0.004
	new	3.306	4.043	4.284	4.449	4.474	0.014
CCSD		3.295	4.025	4.274	4.447	4.458	

a Mean absolute error is also reported. With full space approximate doubles, the resulting active space contains *n*_o_ = 79 occupied and *n*_v_ = 527 virtual orbitals. With reduced space approximate doubles, the resulting active space contains *n*_o_ = 75 occupied and *n*_v_ = 507 virtual orbitals. In total, the molecule has 83 occupied orbitals and 866 virtual orbitals (excluding the frozen core orbitals).

**Figure 5:**
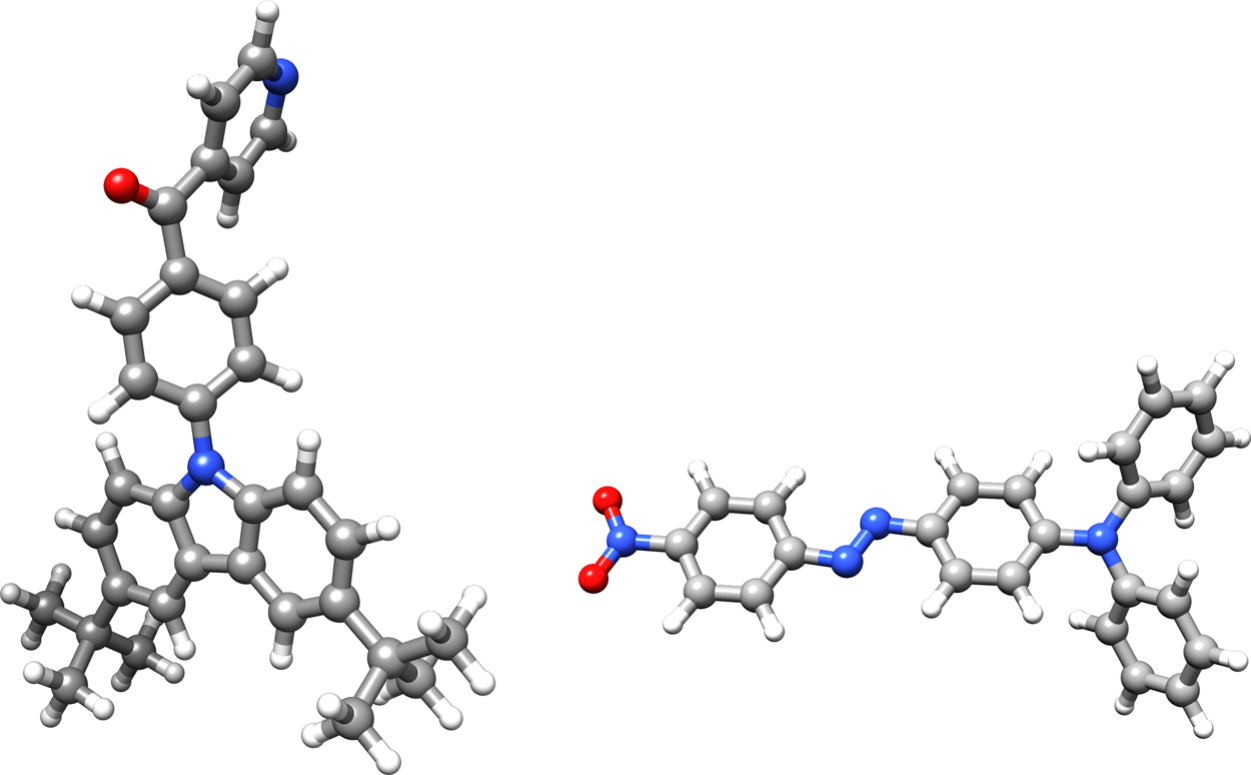
On the left: BPy-pTC^44^ and on the right: an azobenzene dye.

Finally, we have used the new CNTOs to calculate the lowest five excited states (singlet and triplet) of two additional molecules (see Figure 5). The CNTO thresholds are δ_o_, δ_v_ = 10^–4^. The results are compared to the standard coupled cluster models: CCS, CC2, and CCSD. The results can be found in Tables 3–6. The geometry of BPy-pTC can be found in the Supporting Information of ref (40), and the geometry of the azobenzene dye can be found in ref (43). Also for these systems, the mean absolute errors (compared to CC2 and CCSD calculations) are on the order of 0.1 eV or smaller. The dominant CCS NTOs for these two molecules can also be found in the Supporting Information*.* As for the CzTDF molecule, the excitations vary in their degree of localization within the molecule.

**Table 3:** Five Lowest MLCC2/aug-cc-pVDZ and MLCCSD/aug-cc-pVDZ Triplet Excitation Energies of BPy-pTC in eV, Compared to the Standard Coupled Cluster Models^a^

model	ω_1_	ω_2_	ω_3_	ω_4_	ω_5_	MAE
CCS	3.0445	3.1469	3.4356	3.6033	4.2057	
MLCC2	3.1971	3.3495	3.5809	3.7169	4.0315	0.015
CC2	3.1967	3.3962	3.5588	3.7201	4.0804	
MLCCSD	3.2800	3.4513	3.4842	3.6547	4.0450	0.022
CCSD	3.3047	3.4563	3.5491	3.6591	4.0539	

a Mean absolute error is also reported. The resulting active space contains *n*_o_ = 72 occupied and *n*_v_ = 344 virtual orbitals. In total, the molecule has 88 occupied orbitals and 967 virtual orbitals (excluding the frozen core orbitals).

**Table 4:** Five Lowest MLCC2/aug-cc-pVDZ and MLCCSD/aug-cc-pVDZ Singlet Excitation Energies of BPy-pTC in eV, Compared to the Standard Coupled Cluster Models^a^

model	ω_1_	ω_2_	ω_3_	ω_4_	ω_5_	MAE
CCS	4.8324	5.0136	5.1082	5.1397	5.4514	
MLCC2	3.5227	3.8146	3.8903	4.3241	4.4231	0.044
CC2	3.4811	3.7116	3.8665	4.2906	4.4035	
MLCCSD	3.9594	4.0888	4.1386	4.6016	4.7188	0.021
CCSD	3.8884	4.0736	4.1336	4.5912	4.7168	

a Mean absolute error is also reported. The resulting active space contains *n*_o_ = 75 occupied and *n*_v_ = 491 virtual orbitals. In total, the molecule has 88 occupied orbitals and 967 virtual orbitals (excluding the frozen core orbitals).

**Table 5:** Five Lowest MLCC2/aug-cc-pVDZ and MLCCSD/aug-cc-pVDZ Triplet Excitation Energies of the Azobenzene Dye in eV, Compared to the Standard Coupled Cluster Models^a^

model	ω_1_	ω_2_	ω_3_	ω_4_	ω_5_	MAE
CCS	2.0697	2.2356	3.1716	3.2564	3.4086	
MLCC2	2.1603	2.2788	3.1982	3.4723	3.7338	0.092
CC2	2.1766	2.2393	3.4438	3.6615	3.7814	
MLCCSD	2.2187	2.3113	3.2783	3.4212	3.6097	0.040
CCSD	2.2312	2.3001	3.3910	3.5247	3.5905	

a Mean absolute error is also reported. The resulting active space contains *n*_o_ = 65 occupied and *n*_v_ = 324 virtual orbitals. In total, the molecule has 73 occupied orbitals and 749 virtual orbitals (excluding the frozen core orbitals).

**Table 6:** Five Lowest MLCC2/aug-cc-pVDZ and MLCCSD/aug-cc-pVDZ Singlet Excitation Energies of the Azobenzene Dye in eV, Compared to the Standard Coupled Cluster Models^a^

model	ω_1_	ω_2_	ω_3_	ω_4_	ω_5_	MAE
CCS	3.3919	3.9007	5.0663	5.3009	5.3633	
MLCC2	2.6938	2.7267	3.8717	4.1884	4.2999	0.079
CC2	2.6802	2.7301	3.8372	3.8808	4.2595	
MLCCSD	2.8822	3.2969	4.1225	4.2503	4.5781	0.045
CCSD	2.8865	3.2936	4.0507	4.1060	4.5672	

a Mean absolute error is also reported. The resulting active space contains *n*_o_ = 67 occupied and *n*_v_ = 455 virtual orbitals. In total, the molecule has 73 occupied orbitals and 749 virtual orbitals (excluding the frozen core orbitals).

## Conclusions

We propose a procedure with asymptotic -scaling to obtain approximate CNTOs for MLCC2 and MLCCSD calculations. We use reduced-space approximate double excitation vectors to generate the CNTOs. The resulting active space is compact and suitable for valence excitations with MLCC2 or MLCCSD. Errors compared to CC2 and CCSD are well within 0.1 eV using CNTO thresholds of 10^–4^; this is the target accuracy of MLCC2 and MLCCSD excited states, as it is also the typical error in CC2 and CCSD valence excitation energies.

## Supporting Information Available

Dominant NTOs (CCS) for CzTDF, BPy-pTC, and the azobenzene dye molecules (PDF)
