# Supplementary material for: Reduced Scaling Correlated Natural Transition Orbitals for Multilevel Coupled Cluster Calculations
Source: J Phys Chem A. 2024 Oct 24;128(44):9688–94. doi: 10.1021/acs.jpca.4c06271 (PMC11551955; doi:10.1021/acs.jpca.4c06271)
Supplement: Supplementary file 1 — jp4c06271_si_001.pdf [file jp4c06271_si_001.pdf]

Supporting Information for "Reduced Scaling Correlated Natural Transition Orbitals for Multilevel Coupled Cluster Calculations“

Sarai Dery Folkestad* and Henrik Koch

Department of Chemistry, Norwegian University of Science and Technology, NTNU, 7491 Trondheim, Norway

E-mail: sarai.d.folkestad@ntnu.no

CCS natural transition orbitals (NTOs) for CzTDF

Table S1: CCS excitation energies (ω) and NTO eigenvalues (σ) $> 10^{-1}$ for the first 5 singlet excitations of CzTDF

Excitation	ω [eV]	σ [a.u.]	
1	3.8296	0.89	
2	5.0764	0.73	0.19
3	5.1606	0.76	0.14
4	5.2190	0.71	0.18
5	5.3711	0.54	0.43

Table S2: CCS excitation energies (ω) and NTO eigenvalues (σ) $> 10^{-1}$ for the first 5 triplet excitations of CzTDF

Excitation	ω [eV]		σ [a.u.]	
1	1.9830	0.84		
2	3.0213	0.53	0.30	
3	3.0655	0.60	0.16	
4	3.6107	0.37	0.19	0.15
5	3.7238	0.41	0.21	

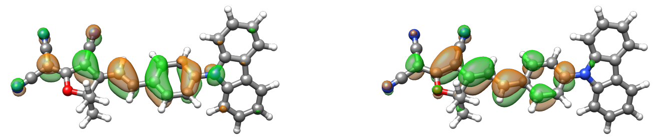

(a) Excitation energy 3.83 eV, NTO eigenvalue 0.89 a.u. Occupied NTO on the left and virtual NTO on the right.

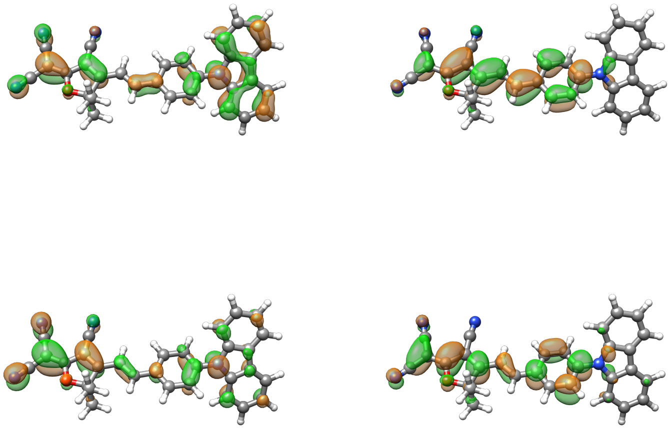

(b) Excitation energy 5.08 eV, NTO eigenvalues: 0.73 a.u. and 0.19 a.u. Occupied NTOs on the left and virtual NTOs on the right.

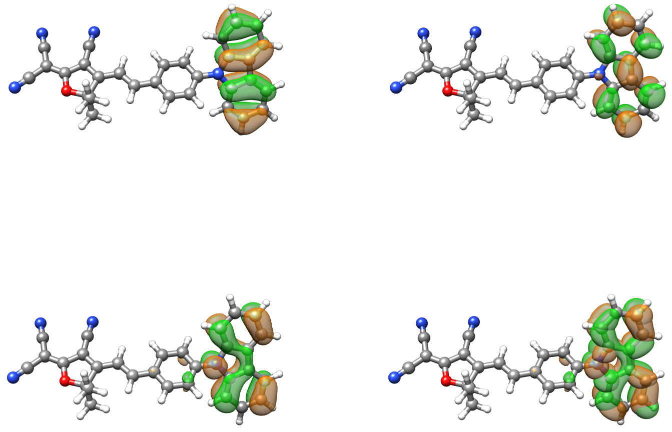

(c) Excitation energy 5.16 eV, NTO eigenvalues: 0.76 a.u. and 0.14 a.u. Occupied NTOs on the left and virtual NTOs on the right.

Figure S1: Most important CCS/aug-cc-pVDZ NTOs for the three lowest singlet excitations of CzTDF. Orbitals are plotted with isovalue 0.03 a.u. Occupied NTOs on the left and virtual NTOs on the right.

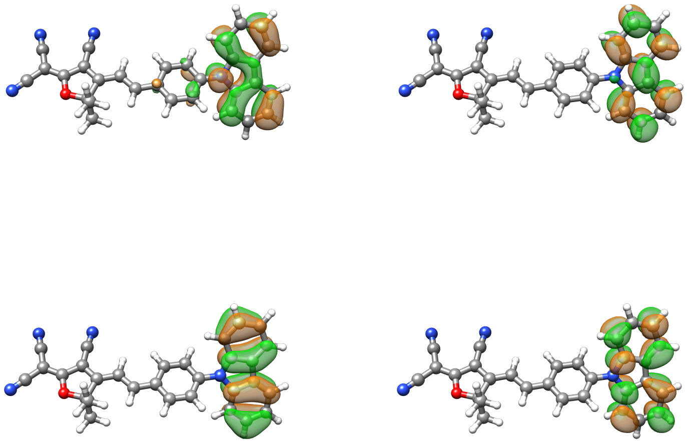

(a) Excitation energy 5.22 eV, NTO eigenvalues: 0.71 a.u. and 0.18 a.u. Occupied NTOs on the left and virtual NTOs on the right.

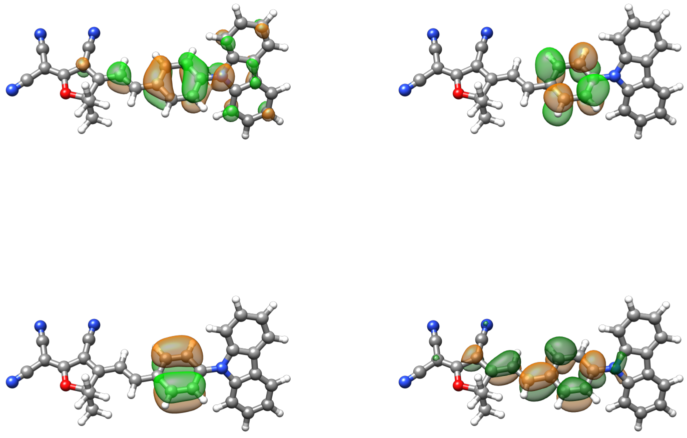

(b) Excitation energy 5.37 eV, NTO eigenvalues: 0.54 a.u. and 0.43 a.u. Occupied NTOs on the left and virtual NTOs on the right.

Figure S2: Most important CCS/aug-cc-pVDZ NTOs for the fourth and fifth singlet excitation of CzTDF Orbitals are plotted with isovalue 0.03 a.u. Occupied NTO are shown in the left column and virtual NTOs are shown in the right column.

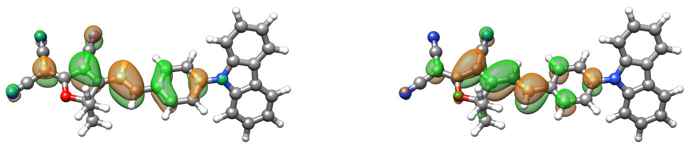

(a) Excitation energy 1.9830 eV, NTO eigenvalue: 0.84 a.u. Occupied NTO on the left and virtual NTO on the right.

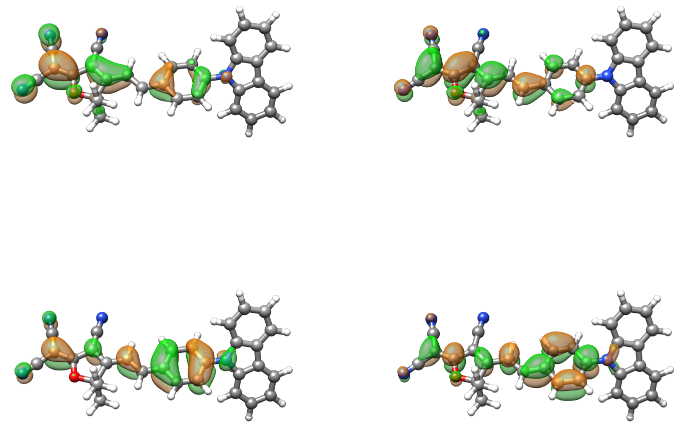

(b) Excitation energy 3.0213 eV, NTO eigenvalues: 0.53 a.u. and 0.30 a.u. Occupied NTOs on the left and virtual NTOs on the right.

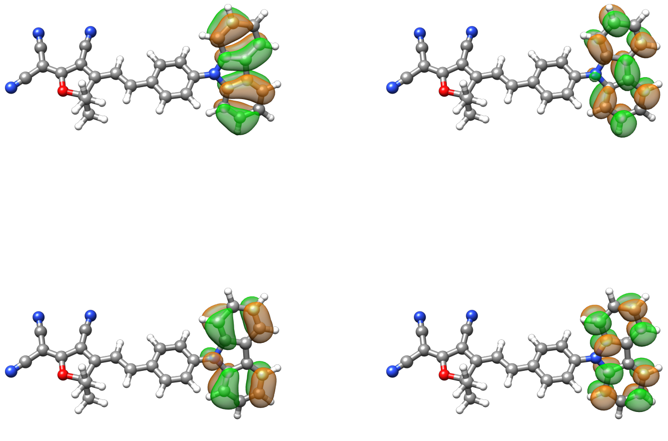

(c) Excitation energy 3.0655 eV, NTO eigenvalues: 0.60 a.u. and 0.16 a.u. Occupied NTOs on the left and virtual NTOs on the right.

Figure S3: Most important CCS/aug-cc-pVDZ NTOs for the three lowest triplet excitations of CzTDF. Orbitals are plotted with isovalue 0.03 a.u. Occupied NTOs on the left and virtual NTOs on the right.

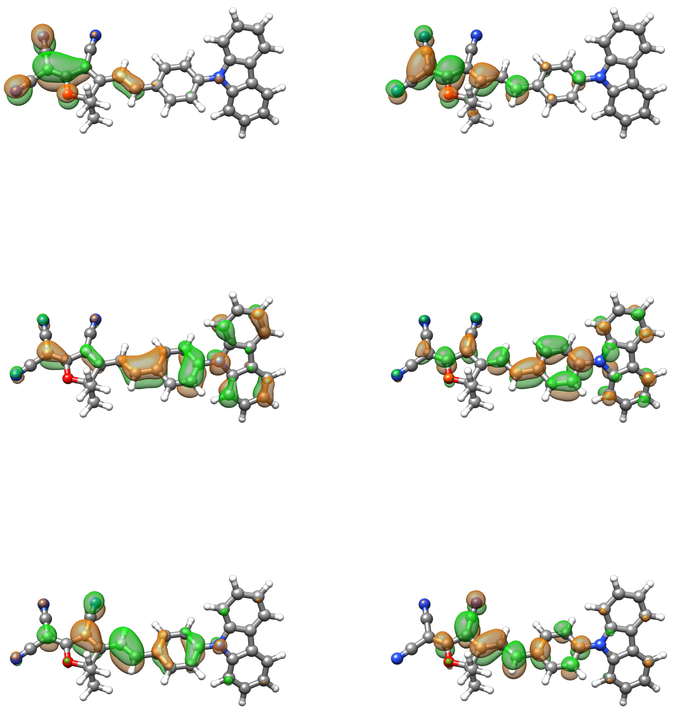

(a) Excitation energy 3.6107 eV, NTO eigenvalues: 0.37 a.u., 0.19 a.u., and 0.15 a.u. Occupied NTOs on the left and virtual NTOs on the right.

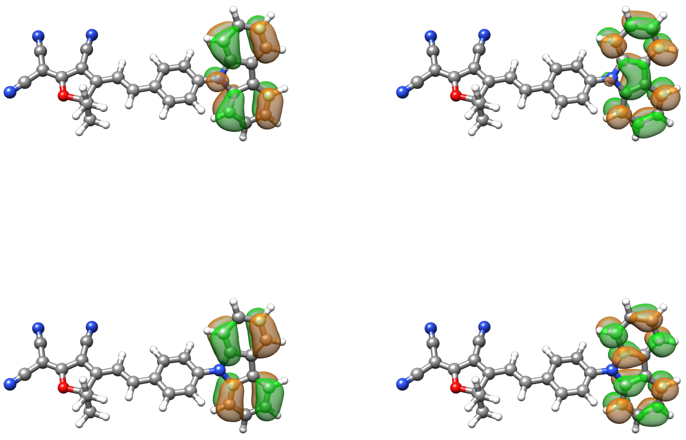

(b) Excitation energy 3.7238 eV, NTO eigenvalues: 0.41 a.u. and 0.21 a.u. Occupied NTOs on the left and virtual NTOs on the right.

Figure S4: Most important CCS/aug-cc-pVDZ NTOs for the fifth triplet excitation of CzTDF Orbitals are plotted with isovalue 0.03 a.u. Occupied NTOs on the left and virtual NTOs on the right.

CCS natural transition orbitals (NTOs) for BPy-pTC

Table S3: CCS excitation energies (ω) and NTO eigenvalues (σ) $> 10^{-1}$ for the first 5 triplet excitations of BPy-pTC

Excitation	ω [eV]	σ [a.u.]		
1	3.0445	0.57	0.19	0.11
2	3.1469	0.67	0.20	
3	3.4356	0.50	0.38	
4	3.6033	0.56	0.26	
5	4.2057	0.75	0.22	

Table S4: CCS excitation energies (ω) and NTO eigenvalues (σ) $> 10^{-1}$ for the first 5 singlet excitations of BPy-pTC

Excitation	ω [eV]	σ [a.u.]	
1	4.8324	0.95	
2	5.0136	0.81	
3	5.1082	0.74	0.13
4	5.1397	0.76	0.14
5	5.4514	0.82	

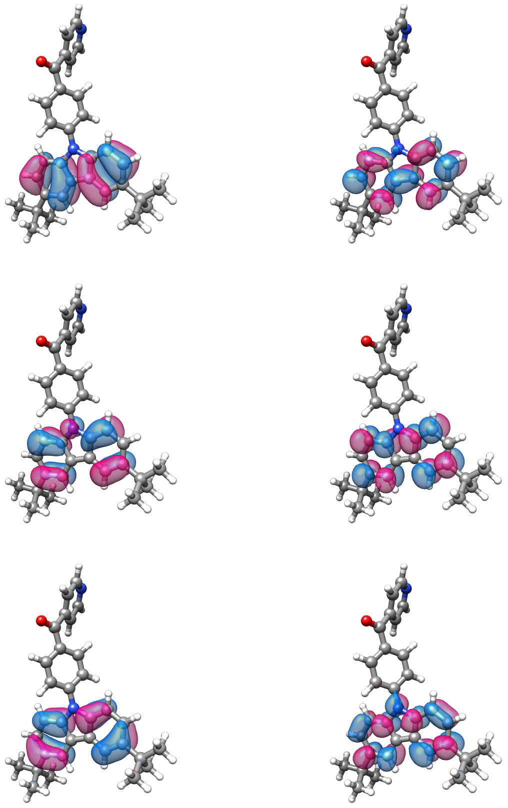

(a) Excitation energy 3.04 eV, NTO eigenvalues: 0.57 a.u., 0.19 a.u. and 0.11 a.u. Occupied NTOs on the left and virtual NTOs on the right.

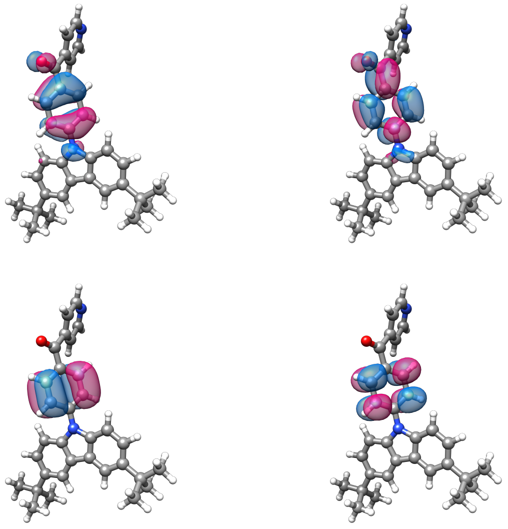

(b) Excitation energy 3.15 eV, NTO eigenvalues: 0.67 a.u. and 0.20 a.u. Occupied NTOs on the left and virtual NTOs on the right.

Figure S5: Most important CCS/aug-cc-pVDZ NTOs for the first and second triplet excitations of BPy-pTC. Orbitals are plotted with isovalue 0.03 a.u. Occupied NTOs on the left and virtual NTOs on the right.

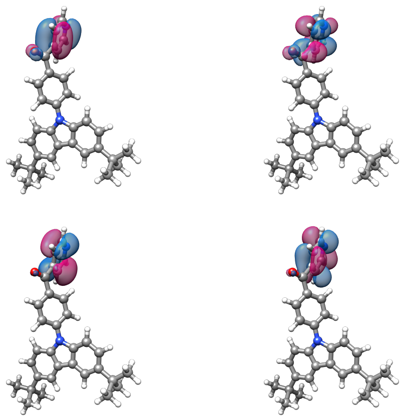

(a) Excitation energy 3.44 eV, NTO eigenvalues: 0.50 a.u. and 0.38 a.u. Occupied NTOs on the left and virtual NTOs on the right.

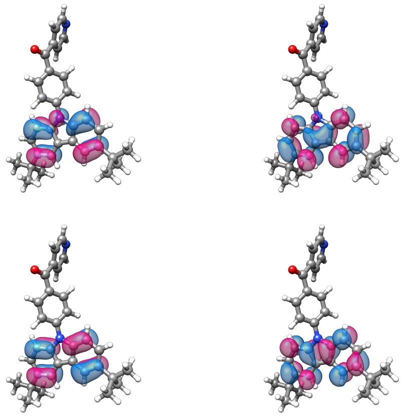

(b) Excitation energy 3.60 eV, NTO eigenvalues: 0.576 a.u., 0.26 a.u. and 0.11 a.u. Occupied NTOs on the left and virtual NTOs on the right.

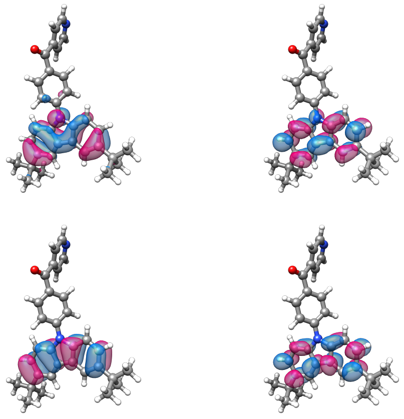

(c) Excitation energy 4.21 eV, NTO eigenvalues: 0.75 a.u. and 0.22 a.u. Occupied NTOs on the left and virtual NTOs on the right.

Figure S6: Most important CCS/aug-cc-pVDZ NTOs for the third, fourth, and fifth triplet excitations of BPy-pTC. Orbitals are plotted with isovalue 0.03 a.u. Occupied NTOs on the left and virtual NTOs on the right.

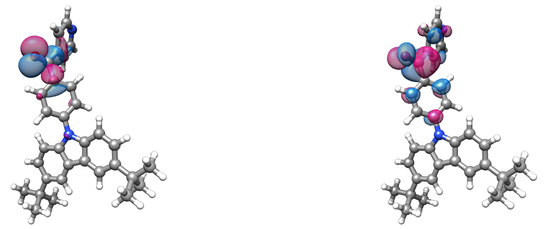

(a) Excitation energy 4.83 eV, NTO eigenvalue 0.95 a.u. Occupied NTO on the left and virtual NTO on the right.

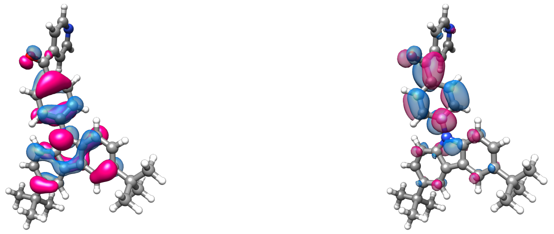

(b) Excitation energy 5.01 eV, NTO eigenvalues: 0.81 a.u. Occupied NTOs on the left and virtual NTOs on the right.

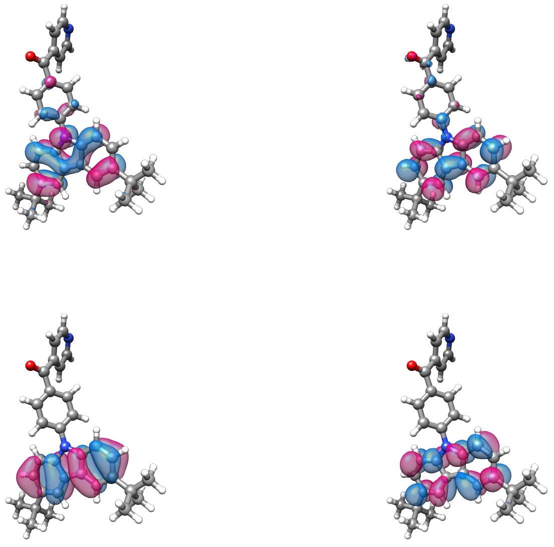

(c) Excitation energy 5.11 eV, NTO eigenvalues: 0.74 a.u. and 0.13 a.u. Occupied NTOs on the left and virtual NTOs on the right.

Figure S7: Most important CCS/aug-cc-pVDZ NTOs for the three lowest singlet excitations of BPy-pTC. Orbitals are plotted with isovalue 0.03 a.u. Occupied NTOs on the left and virtual NTOs on the right.

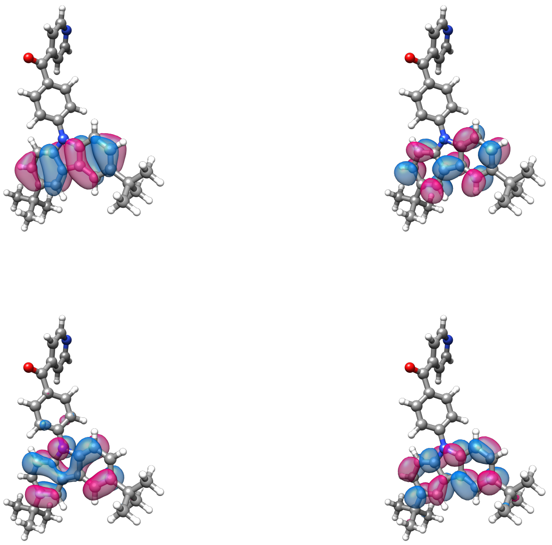

(a) Excitation energy 5.14 eV, NTO eigenvalues: 0.76 a.u. and 0.14 a.u. Occupied NTOs on the left and virtual NTOs on the right.

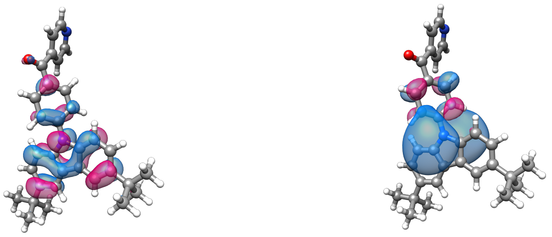

(b) Excitation energy 5.11 eV, NTO eigenvalues: 0.82 a.u. Occupied NTOs on the left and virtual NTOs on the right. The virtual NTO is plotted with an isovalue of 0.02 a.u.

Figure S8: Most important CCS/aug-cc-pVDZ NTOs for the fourth and fifth singlet excitations of BPy-pTC. Orbitals are plotted with isovalue 0.03 a.u., unless otherwise stated. Occupied NTOs on the left and virtual NTOs on the right.

CCS natural transition orbitals (NTOs) for the azobenzene dye

Table S5: CCS excitation energies (ω) and NTO eigenvalues (σ) > 10^{-1} for the first 5 triplet excitations of the azobenzene dye

Excitation	ω [eV]	σ [a.u.]			
1	2.0697	0.82			
2	2.2356	0.99			
3	3.1716	0.39	0.27	0.12	
4	3.2564	0.42	0.19	0.19	0.15
5	3.4086	0.24	0.21	0.17	0.16

Table S6: CCS excitation energies (ω) and NTO eigenvalues (σ) > 10^{-1} for the first 5 singlet excitations of the azobenzene dye

Excitation	ω [eV]	σ [a.u.]	
1	3.3919	0.99	
2	3.9007	0.89	
3	5.0663	0.74	0.14
4	5.3009	0.49	0.35
5	5.3633	0.79	

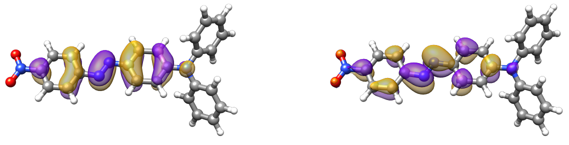

(a) Excitation energy 2.07 eV, NTO eigenvalue 0.82 a.u. Occupied NTO on the left and virtual NTO on the right.

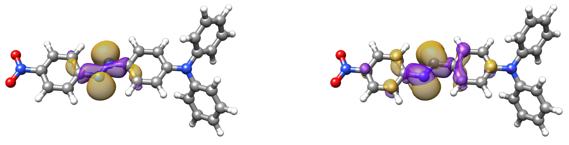

(b) Excitation energy 2.24 eV, NTO eigenvalues: 0.99 a.u. Occupied NTOs on the left and virtual NTOs on the right.

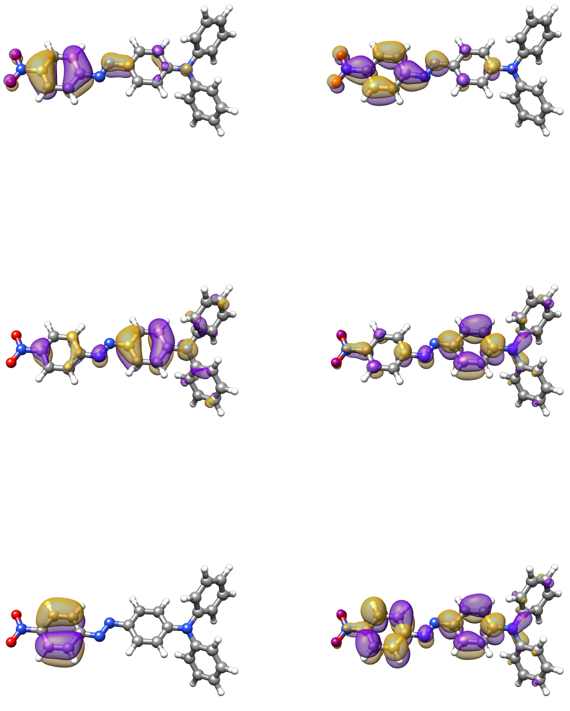

(c) Excitation energy 3.17 eV, NTO eigenvalues: 0.39 a.u., 0.27 a.u. and 0.12 a.u. Occupied NTOs on the left and virtual NTOs on the right.

Figure S9: Most important CCS/aug-cc-pVDZ NTOs for the three lowest triplet excitations of the azobenzene dye. Orbitals are plotted with isovalue 0.03 a.u. Occupied NTOs on the left and virtual NTOs on the right.

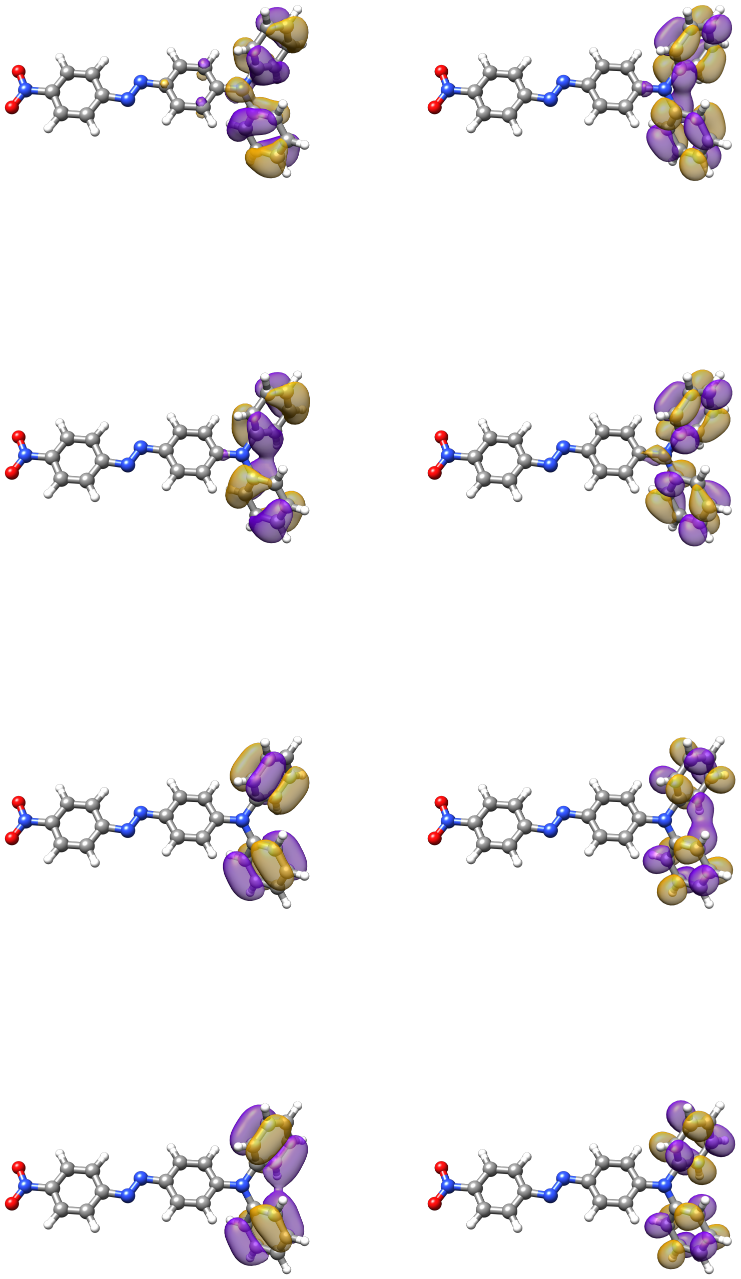

Figure S10: Most important CCS/aug-cc-pVDZ NTOs for the fourth triplet excitations of the azobenzene dye. Excitation energy 3.26 eV, NTO eigenvalue 0.42 a.u., 0.19 a.u., 0.19 a.u. and 0.15 a.u.Occupied NTO on the left and virtual NTO on the right. Orbitals are plotted with isovalue 0.03 a.u. Occupied NTOs on the left and virtual NTOs on the right.

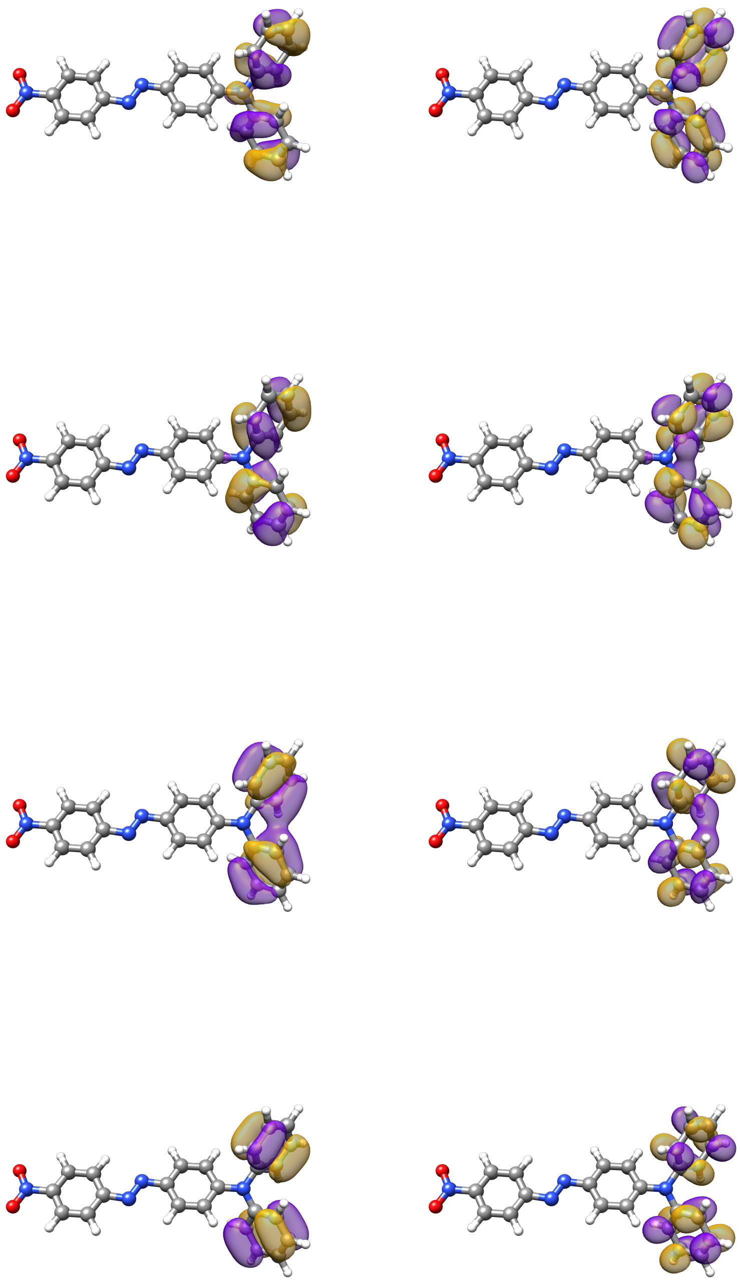

Figure S11: Most important CCS/aug-cc-pVDZ NTOs for the fifth triplet excitations of the azobenzene dye. Excitation energy 3.41 eV, NTO eigenvalue 0.24 a.u., 0.21 a.u., 0.17 a.u. and 0.16 a.u.Occupied NTO on the left and virtual NTO on the right. Orbitals are plotted with isovalue 0.03 a.u. Occupied NTOs on the left and virtual NTOs on the right.

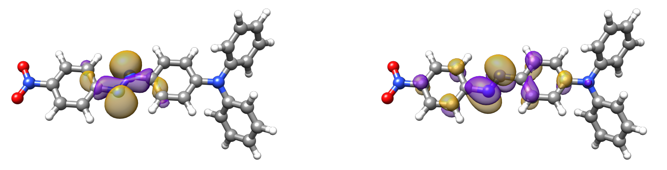

(a) Excitation energy 3.39 eV, NTO eigenvalue 0.99 a.u. Occupied NTO on the left and virtual NTO on the right.

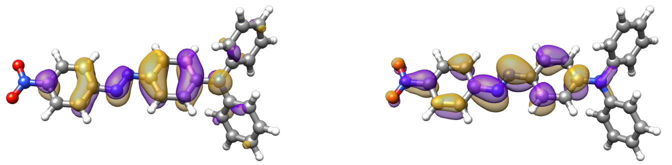

(b) Excitation energy 3.90 eV, NTO eigenvalues: 0.89 a.u. Occupied NTOs on the left and virtual NTOs on the right.

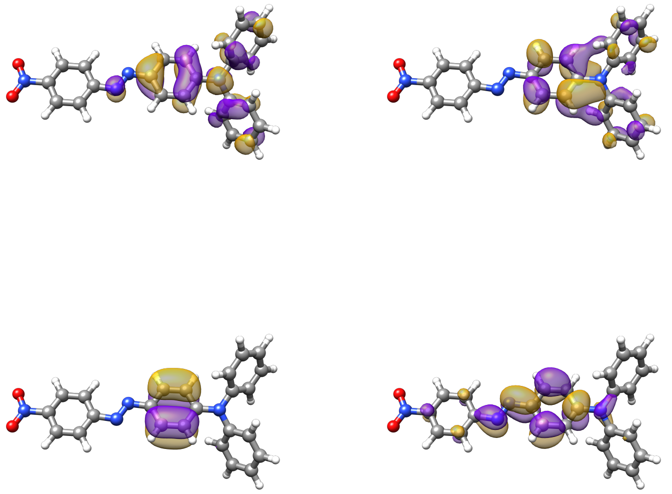

(c) Excitation energy 5.07 eV, NTO eigenvalues: 0.74 a.u. and 0.14 a.u. Occupied NTOs on the left and virtual NTOs on the right.

Figure S12: Most important CCS/aug-cc-pVDZ NTOs for the three lowest singlet excitations of the azobenzene dye. Orbitals are plotted with isovalue 0.03 a.u. Occupied NTOs on the left and virtual NTOs on the right.

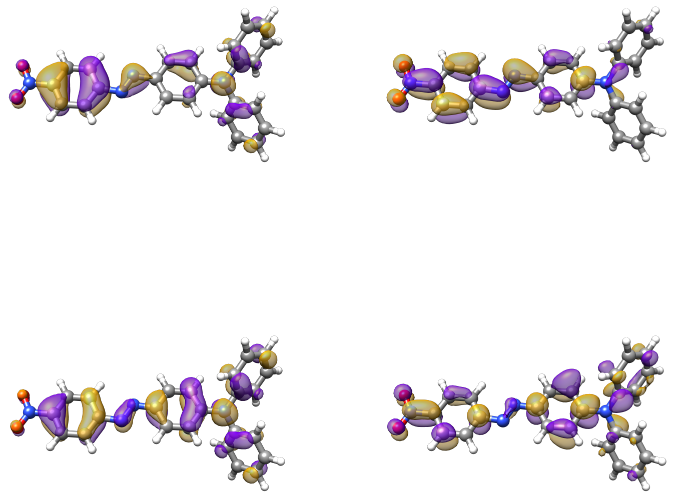

(a) Excitation energy 5.30 eV, NTO eigenvalue 0.49 a.u. and 0.35 a.u.Occupied NTO on the left and virtual NTO on the right.

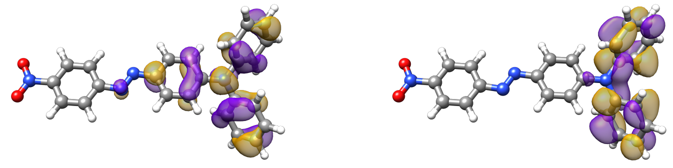

(b) Excitation energy 5.36 eV, NTO eigenvalues: 0.79 a.u. Occupied NTOs on the left and virtual NTOs on the right.

Figure S13: Most important CCS/aug-cc-pVDZ NTOs for the fourth and fifth singlet excitations of the azobenzene dye. Orbitals are plotted with isovalue 0.03 a.u. Occupied NTOs on the left and virtual NTOs on the right.
